# Genetic Regulatory Networks of Apolipoproteins and Associated Medical Risks

**DOI:** 10.3389/fcvm.2021.788852

**Published:** 2022-01-06

**Authors:** Preethi Basavaraju, Rubadevi Balasubramani, Divya Sri Kathiresan, Ilakkiyapavai Devaraj, Kavipriya Babu, Vasanthakumar Alagarsamy, Vinayaga Moorthi Puthamohan

**Affiliations:** ^1^Biomaterials and Nano-Medicine Laboratory, Department of Human Genetics and Molecular Biology, Bharathiar University, Coimbatore, India; ^2^Department of Human Genetics and Molecular Biology, Bharathiar University, Coimbatore, India

**Keywords:** apolipoproteins, polymorphisms, haplotypes, medical complications, global population

## Abstract

Apolipoproteins (APO proteins) are the lipoprotein family proteins that play key roles in transporting lipoproteins all over the body. There are nearly more than twenty members reported in the APO protein family, among which the A, B, C, E, and L play major roles in contributing genetic risks to several disorders. Among these genetic risks, the single nucleotide polymorphisms (SNPs), involving the variation of single nucleotide base pairs, and their contributing polymorphisms play crucial roles in the apolipoprotein family and its concordant disease heterogeneity that have predominantly recurred through the years. In this review, we have contributed a handful of information on such genetic polymorphisms that include *APOE*, ApoA1/B ratio, and *A1/C3/A4/A5* gene cluster-based population genetic studies carried throughout the world, to elaborately discuss the effects of various genetic polymorphisms in imparting various medical conditions, such as obesity, cardiovascular, stroke, Alzheimer's disease, diabetes, vascular complications, and other associated risks.

## Introduction

### Apolipoproteins

Apolipoproteins (APO proteins) are the major class of proteins that play vital roles in the binding and transport of lipid molecules to form lipoproteins (1). There are six major classes of APO proteins: A, B, C, D, E, and H and F and G (2). The human genome encodes for nearly more than 21 *APO* genes, out of which *APOA (APOA1, APOA2, APOA4*, and *APOA5), APOC (APOC1, APOC2*, and *APOC3), APOB*, and *APOE (APOE2, APOE3*, and *APOE4)* contribute to coding proteins that regulate triglycerides (TGs) and cholesterol metabolism, transport, and management at cellular levels (3–5). The genetic variations studied in these coding genes have a wide range of contributions toward the medical conditions that provoke a phenomenon known as ‘multiple heterogeneities (6). The genetic studies allow us to unravel the aetiological heterogeneity of specific gene polymorphisms at a molecular level to understand its genotype variations and concordant risks associated with several disease conditions throughout the genome (7). In the present review, we have shed light on the major polymorphisms and mutations that are widely studied as genotypic expressions associated with the different *APO* genes, linked to a wide range of disease severities and risks (8).

The major genetic risks associated with the different APO proteins are listed in Table 1. Analysis of genetic variations predominantly involves the screening of single nucleotide polymorphisms (SNPs) residing along the upstream regions of the gene (9). The APO gene family polymorphisms are linked to several diseases, such as *APOE* genotypes with circulating lipid levels and coronary heart risks, cardiovascular disease (CVD), and with the ε4 homozygous genotypes showing increased risk of Alzheimer's disease (AD) (OMIM: 104310), *APOA5* variants and the hypertriglyceridemia (OMIM: 145750), *APOA* polymorphisms with familial hypercholesterolemia (OMIM: 144010), *APOB* gene alterations, and ischemic heart disease (OMIM: 601367) increased risks (10–13).

**Table 1:** The different lipoproteins expressed and their major genotypic risks associated with concordant disease conditions.

**Lipoprotein**	**Major lipids**	**Major apolipoproteins**	**Clinical risks associated**
Chylomicrons	Exogenous triglycerides (TG)	A-I, B-18, C-I, C-II, C-III	Eruptive xanthomas, Hepatosplenomegaly, Pancreatitis
VLDL	Endogenous TG	B-100, C-I, C-II, C-III, E	Pancreatitis, CHD, PVD
IDL	Endogenous TG, cholesterol esters	B-100, E	Multiple myeloma
LDL	Cholesterol esters, free cholesterol	B-100	Tendon xanthomas, CHD
HDL	Phospholipids, cholesterol esters	A-I, A-II	Diabetes, CVD
HDL	Cholesterol esters, TG	L-I	Nephropathies

*HDL, high-density lipoprotein; IDL, intermediate-density lipoprotein; LDL, low-density lipoprotein; VLDL, very low-density lipoprotein; CHD, coronary heart disease; CVD, cardiovascular disease; PVD, peripheral vascular disease (9)*.

Several studies on the genetic polymorphisms of the APO genes have been reported to date, but we do largely shortfall on the cumulative data that on the evident support of future understanding of molecular backgrounds and their contributions toward the aetiological heterogeneity of various diseases linked with APO gene family metabolism and expression. In the current review, we have gathered cumulative information on the various APO gene family-based genotype and polymorphism studies scattered into pieces on population-based criteria to review the backgrounds of the APO gene family polymorphisms and their risks for various clinical conditions all over the world.

### Apolipoprotein A1/C3/A4/A5 Gene Cluster

The plasma lipoprotein segregates into seven classes, namely, the chylomicrons, chylomicron remnants, very low-density lipoprotein (VLDL), intermediate-density lipoprotein (IDL), low-density lipoprotein (LDL), high-density lipoprotein (HDL), and lipoprotein (a) [Lp (a)], among which the HDL levels are designated to be ~1.063–1.21 g/ml as the control parameter, and it mainly comprises of diverse groups of tiny spherical disc shaped particles classified based on their size, charge, density, electrophoretic mobility, and Apolipoprotein compositions (14). The dense composition of the APO proteins comprises the APOA1 that is primarily synthesized in the liver and small intestine (15), playing key roles in the signaling of reverse cholesterol transport mechanisms. Apart from the APOA1, the HDL family proteins comprise the ApoA-II, ApoA-IV, ApoA-V, ApoC-I, ApoC-II, ApoC-III, ApoD, ApoE, ApoJ, ApoL, and ApoM (16).

*Apolipoprotein A4 (APOA4)* is one of the first identified protein components of chylomicrons and HDL family (17, 18) whose physiological role remains unclear, but some of the recent studies have insisted their protective effects against the formation of diet-induced aortic lesions in mice models (19). *APOA5* is the newly identified member of the apolipoprotein gene family by the comparison of human and mouse DNA sequences (20). It is expressed predominantly in the liver, playing crucial roles in maintaining the TG levels. The *APOC3* is another protein component that belongs to the TG-rich lipoproteins and HDL family, which is mainly synthesized in the liver and intestine (21), playing major physiological roles in inhibiting lipoprotein lipases (LPLs).

**Figure 1:**
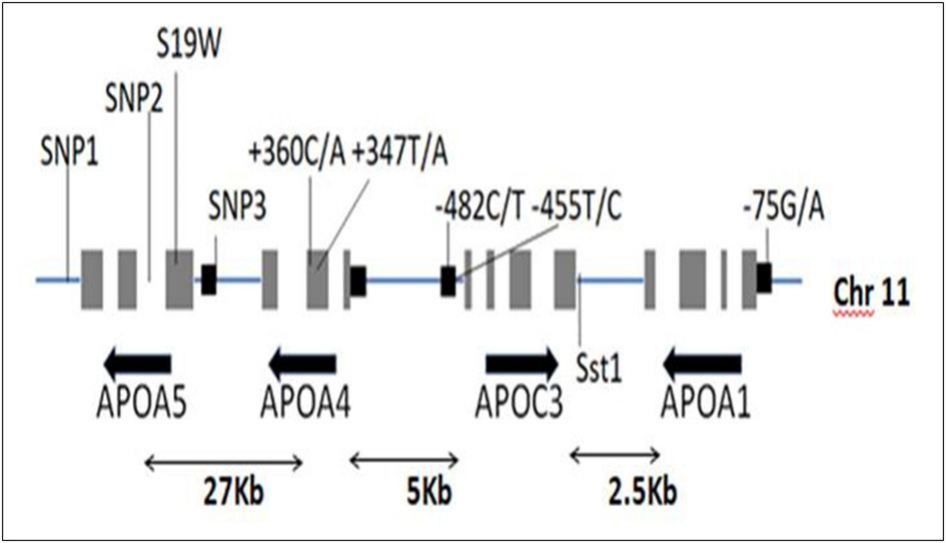
The *APOA1/C3/A4/A5* gene cluster residing on chromosome 11 spanning various polymorphisms.

These APO proteins ApoA-I, ApoA-IV, ApoA-V, and ApoC-III are coded by the *APOA1* (OMIM: 107680), *APOA4* (OMIM: 107690), *APOA5* (OMIM: 606368), and *APOC3* (OMIM: 107720) genes that collectively contribute to the A1/C3/A4/A5 complex which is an incomplete gene cluster with numerous interactions transcribing different factors functioning as immediate upstream regions of each adjacent genes located on the chromosome 11. This gene cluster contributes to ~38% of major genetic mutations that lead to plasma lipoprotein alternating levels (22). The cluster extends up to 17 Kb lengths, where the *APOA1* and *APOA4* genes are transcribed along the same direction, and the *APOC3* gene is transcribed in the opposite direction (20) as shown in Figure 1. Almost at a distance of 30 kb upstream along this cluster is the *APOA5* gene, transcribed through the same direction of *APOA1* and *APOA4* genes. The entire gene cluster covers ~60 kb, comprising of the strong Linkage disequilibrium and polymorphism sites at different loci (3). Three of the four genes belonging to this cluster contain polymorphisms within the upstream region of the gene and are described as the promoter region specific SNPs exhibiting strong associations with several lipid-based alternative variants (20).

**Figure 2:**
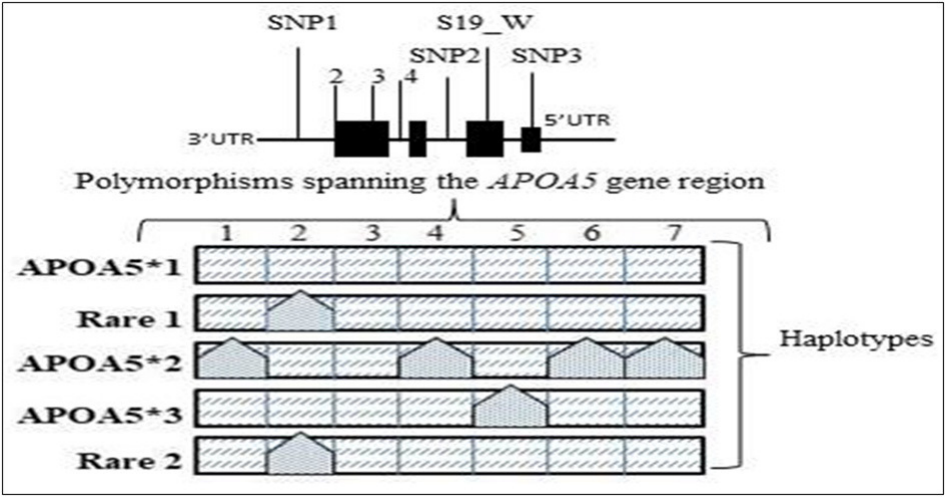
The *APOA5* variants and common haplotypes: specific APOA5 polymorphisms found on the genetic interval contributing to the five major haplotypes seen in various populations worldwide.

The Apolipoprotein family encoding gene cluster and associated enzyme based phenotypic expressions are believed to be one of the underlying mechanisms for maintaining meticulous actions, such as antioxidant, anti-inflammatory, and anti-thrombotic effects (23). The plasma levels of Apo-B and Apo-C (III) are the key predictors of risk factors associated with coronary heart diseases (24), and whereas the ApoB levels alone are interlinked to the intermediate interactions involved in LDL and arterial wall pressure mechanisms, thereby infuriating the development of atherosclerosis conditions at increased levels (25, 26). Hence, the genetic mutations screened within the *APOA1/C3/A4/A5* gene cluster are extensively examined in concordance to the biochemical alterations of serum lipid levels and cardiovascular risks (27–29). The major polymorphisms of the gene cluster span for seven major SNPs give rise to the haplotype alterations, as shown in Table 2 and Figure 2.

**Table 2:** The *APOA5* gene polymorphisms that contribute to the nucleotide variations in the five major haplotypes.

**Polymorphism**	**Variation description**	**Position**	**Haplotype**				
			APOA5*1	APOA5*2	APOA5*3	**Rare 1**	**Rare 2**
1891T>C or 1259T>C	SNP1 rs2266788	3'UTR	T	C	T	T	T
1764C>T	rs619054	3'UTR Stop codon region	C	C	C	T	C
V153M	rs3135507	Coding region	C	C	C	C	T
751G>T or IVS3+476G>A	SNP2 rs2072560	Non-coding region	G	A	G	G	G
c.56C>G or ser19-to-trp	S19_W rs3135506	Coding region	C	C	G	C	C
−3A>G	rs651821	5'UTR Start Codon region	A	G	A	A	A
−1131T>C	SNP3 rs662799	Promotor region	T	C	T	T	T

*A, T, C, and G, nucleotide bases; SNP, single nucleotide polymorphism*.

*The rare type of haplotypes mentioned here are with respect to the number of mentions in the genetic studies so far reported and not the frequency levels*.

*All the variant specifications mentioned are from the asia.ensembl.org/Homo~sapiens/Variation*.

### Apolipoprotein A1/C3/A4/A5 Gene Cluster Polymorphisms and Associated Risks

The gene that codes for the APOA1 protein has *APOA1* 276 → G/A point mutation widely seen in the promoter region and is associated with altered postprandial lipid metabolism defects (30, 31). Apart from this major mutation, the SNPs *APOA1*−2630G/A, *APOA1*−2803G/A, and −3012A/G are seen in the promoter region and are associated with fasting lipid levels alterations (32).

Some mutations seen in the *APOA5* gene, such as T-1131C and Ser19Trp, are linked to the altered fasting, and postprandial TG levels (33, 34), and this gene also contributes to three main haplotypes based on five polymorphisms by *APOA5, APOA5*, and *APOA5*: 1131T>C, c.3A>G, c.56C>G, IVS3+476G>A, and c.1259T>C, respectively. Several SNPs within *APOC3* promoter regions have been studied for the association with altered TGs levels (35–38), out of which the three SNPs *APOC3* rs5128 (SNP 41) *Sst1*, associated with altered fasting triglycerides, *APOC3* rs2854117 (SNP38)−482C/T, associated with longevity and HDL levels (39). The *APOC3* rs2854116 (SNP 39)−455T/C, is classified as type II and widely studied polymorphs strongly associated with the increased TG levels.

The *APOA1/C3/A4/A5* gene cluster has been assumed to be the roots of various diseases that include TG and TC levels, among them a study reported on English families consisted of 84 known occurrences of familial combined hyperlipidaemia (FCH) subjects and their relative spouses totalling a sample size of ~200 and more were analyzed a span of eight SNPs (*APOA5* 58892C > T, *APOA5* 56C > G, *APOA5* 12238T > C, *APOC3* 386C >G, and *APOA1* 3031C > T) encountered within the gene cluster to show that a single haplotype of the *APOA5* gene caused by a missense mutation was seen 3-folds higher in the FCH probands when compared with the relative spouses. Similarly, the secondary haplotypes of the *APOC3* and *APOA5* genes had relatively higher occurrences in the spouse subjects compared with that of the actual probands (40). Later a similar linkage and association study on 128 Dutch FCH families showed that the two independent alleles of *APOA5* or *APOC3* genes (APOA556C > G and APOC3 386C >G) belonging to the *APOA1/C3/A4/A5* gene cluster were overexpressed in all subjects irrespective of the common haplotype of the *APOA1/C3/A4/A5* gene cluster being expressed at a very low transmission level (41). These results have spotlighted the effects of *APOA1/C3/A4/A5* gene cluster haplotypes that have stronger evidence over the plasma TG and LDL levels, thereby contributing to the known recurrences of the FCH families in an orderly pattern.

An ancestral study on Sri Lankan population comprising of 95 men, screened for *ApoA-I/C-III/A-IV*-SstI associated SNPs showed higher risks for CHD (42), and the reports of African-American type 2 diabetic population consisting of 168 subjects had confirmed APOC III levels alone serve as the major risk factors of metabolic syndromes with decreased TG and HDL levels contributing to the high risks of CHD and dyslipidemia in the Tri-ethnic groups chosen (white Non–Hispanics, African-American, and Hispanics) (43). Similarly, various other studies have reported the plasma and serum levels of the TG molecules and their concordant associations for the risks of CVD, growth hormone deficiency (GHD), and CHD increasingly in APOC3 and APOA5 rs2854116 and rs662799 polymorphisms in the European population but contrast show lower risks for the Triethnic groups (Non–Hispanics, African-American, and Hispanics) in C allele with the higher HDL levels (44–47). Whereas, a study conducted on the American population predominantly comprising of white subjects totalling several ~1,200 men and women with known CVD risks were analyzed for several SNPs harboring within the *APOA1/C3/A4/A5* gene cluster and showed that the *APOA5* gene associated 56C > G and the1,131T > C SNPs were having higher transmission rates and expressed high TG, remnant-like particles, large and intermediate VLDL levels in both men and women CVD subjects (48). In addition, the study concluded with several new haplotype expressions and showed women with the 1131C allele were associated with higher risk assessments of CVD complications.

The gender and ethnicity-based study on Asian women (*n* = 973) having dyslipidaemia complications compared with that of the European women by the same team also showed stronger influences for CVD and CHD risks *via* the *APOA5*T-1131C promoter polymorphism, depicting the minor−1131C allele for both homozygotes and heterozygotes conditions that were associated with elevated plasma TG, VLDL-TG, LDL-TG, HDL-TG, and VLDL-cholesterol levels. Concomitantly, a study on Singapore population comprising of the Chinese, Indian, and Malay groups totalling a sum of ~3,971 subjects screened for the interactions among plasma lipids levels and the *APOA5* polymorphisms showed increased levels of plasma TG and TC in association with the C allele of the *APOA5* polymorphism, where the levels of HDL and HDL-C remained same across the polymorphisms for all the ethnic groups. The study also suggested that the *APOA5* genotypes play major roles in plasma TG, TC, and HDL cholesterol exhibiting ethnic differences (49).

Further, the study on the genetic modulation of serum fasting triacylglycerol levels and the *APOA5* gene alterations had shown the elevation of postprandial triacylglycerol concentrations in ~158 non-obese subjects habituating to the Korean population in association with the *APOA5*T-1131C promoter polymorphism serving to be one of the standard markers of oxidation and inflammation risks leading to major CVD risks. As an insight to add more information on the *APOA1/C3/A4/A5* gene cluster interlinked cardiovascular disorders, *APOC3* genotype based report on Spanish (Costa Dica) population including 336 individuals screened for the *APOC3* [promoter region 455-T > C, T-625del and C3238G 3' untranslated region (UTR)] polymorphisms associated risks for CVD showed no evident interactions among plasma lipoproteins, and saturated fat intake in APOC3-455C-625del allele carriers neither did the 3'UTR C3238G have any observed association for the CVD risks. In contrast, a habitat based low saturated fat diet was found beneficial for the plasma lipoprotein levels among the APOC3 homozygotes of the chosen polymorphisms (50). Another study on the Ely (European) cohort including ~500 individuals screened for two variants of the insulin responsive element (IRE) of the APOC3 promoter region (−455T > C and −482C > T) were in Hardy Weinberg equilibrium, and the differences between the polymorphisms were observed to be less with reduced insulin secretion in male −482T/T carriers followed by the increase in insulin levels later.

The study concluded with milder links between the altered non-esterified fatty acid (NEFA) in men for both the T and C allele of the polymorphism and higher risks of diabetic onsets along with CHD for the *APOC3*−482C/T polymorphism (51). Similarly, the serum LDL cholesterol in men has a strong risk associated with the A allele of *APOA1-*75G/A polymorphism as per the study conducted on ~800 Calabria (southern Italy) population for the risk of CVD whereas, showing a decreased risk of the G allele among the three genotypes screened in the middle aged and elderly population (52). These cumulative results suggest that the risk associated with the increase and decrease of the biochemical markers of the CHD, CVD, and coronary artery disease (CAD) based diseases are not only significantly linked to the corresponding genetic polymorphisms seen in the *APOA1/C3/A4/A5* gene cluster but also have strongly unpinned associations with the aetiological heterogeneity of their habituations.

### Apolipoprotein A1/B Ratio Polymorphisms and Associated Medical Risks

The APO proteins are strong biomarkers of several cardiovascular events, and we have been listing all the major literature that has lined up in the row of finding their roles. Apart from the gene polymorphism studies, the Apolipoprotein A1/B ratio levels in the serum and plasma samples play a vital biochemical role in diagnosing an atherogenic risk of an individual (53). The Apolipoprotein A1/B ratio signifies the equilibrium seen among atherogenic and anti-atherogenic lipoproteins mainly observed in the serum and is estimated to be Apolipoprotein A (mg/dl)/Apolipoprotein B (mg/dl): <0.8 for individuals aged <18 years and 0.7–0.9 for individuals aged >18 years in men and <0.8 for individuals aged <18 years and 0.6–0.8 for individuals aged >18 years in women (54).

The *APOB* gene localized on human chromosome 2 encodes for the protein (Apo) B-100 (UniProt:P04114) mainly constituting of chylomicron apo B-48, LDL apo B-100, and VLDL apo B-100, which are some of the principal plasma protein components used to estimate the LDL and LDL receptor cellular binding and internalization of LDL molecules from the liver and peripheral cells, thereby, playing a crucial role in cholesterol homeostasis mechanisms (55–57). Whereas the APOA1 gene on the human chromosome 11 encodes for a serum and/or plasma protein ApoA-I (UniProt: P02647) that acts in the reverse transport events of lipid and cholesterol molecules from tissues to the liver, promoting excretion of cholesterol through efflux mechanism by acting as a cofactor for lecithin cholesterol acyltransferase (LCAT), a key enzyme that regulates the lipoprotein metabolism (58). The alterations in Apolipoprotein A1/B ratio levels are directly linked to the plasma cholesterol levels and are substantially increased *via* the decreased clearance of the LDL molecules by the un-functional expression of APOB/E receptors (59).

A study comprising of Jing and Han ethnic groups of the Chinese population screened for testing the link between genetic polymorphisms in the dedicator of cytokinesis 7 (DOCK7), one of the trans-membrane pro-protein convertase subtilisin/kexin type 9 (PCSK9) enzyme and serum lipid levels. The study consisted of ~1,869 CVD and control subjects of the middle age groups tested for genotype analysis showing Apo-A1: rs1997947 polymorphism predominantly only in the Jing ethnic group and ApoA1: rs11206517 and rs4846913 only in the Han nationals. The study had Apo-B: rs7552841 polymorphism in both the ethnic groups but Apo-B: rs10889332 only in the Han nationals, and the ApoA1/ApoB ratio polymorphisms showed rs7552841 for Jing and rs4846913 in the Han ethnic groups, respectively. Similarly, *APOA1/B* gene based polymorphisms had significant correlations with the plasma TC, TG, and LDL cholesterol levels thereby, imparting a specific new rare haplotype (G-C-G-C-T-G-C-C-A) that had diminishing effects on TC (4.90 ± 0.86 mmol/L), TG (1.30 (1.08) mmol/L), LDL (2.80 ± 0.40 mmol/L) cholesterol, and APOB (1.02 ± 0.22 g/L) levels alone when compared with that of the other 27 haplotypes seen (60).

Later, a subsequent study by the same author concluded that there is strong evidence to confirm, Han ethnic groups of the Chinese population, including 1,640 individuals, show lower risks for the increased levels of TG [1.37 (1.09) mmol/L), LDL (2.85 ± 0.43 mmol/L), APOB (1.05 ± 0.24 g/L], and serum lipid levels thereby, providing a masking effect for the CVD risks and moreover, this effect is specific for certain haplotype (CAPN3- FRMD5 T-A, T-C, C-A, and C-C) carriers only (61). In line with these results, the haplotype-based studies on the ApoA1/B ratio polymorphisms have shown supporting evidence on CVD/CHD, stroke, and/or other cardiovascular events, diabetes, dyslipidaemia at varying levels of risk issues in human samples of different populations including (Han, Mulao, and Maonan) Chinese population.

A study by Berkinbayev et al. (62) showed that the decreased levels of Apo A1 (136.3 ± 5.5 mg/d) and increased levels of the Apo B (88.98 ± 2.63 mg/d) in CHD individuals (*n* = 448) belonging to the Kazakhs have Apo E levels significantly (< 0.05) lower than that of the healthy Uighurs population (62). The risk of CHD/CVD along with stroke and diabetes showed evident occurrences along with the rare haplotypes: *SRGAP2* rs2483058C-rs2580520G of *ApoA1* and *ApoA1/ApoB* ratio, thereby contributing to the development of breast cancer in the Han population having greater risks over the subjects presenting increased levels of circulating oestrogens. In addition, the study also showed that the rs2483058C-rs2580520G haplotype is associated with an increased risk of dyslipidaemia in the Han and Maonan groups (*n* = 2,444), showing consistent risks of increased serum TC (5.32 ± 1.06 mmol/L), HDL-C (1.71 ± 0.58 mmol/L), ApoA1 (1.22 ± 0.14 g/L), and the ApoA1 to ApoB ratio (1.47 ± 0.47 g/L) (63).

The *APOB* gene polymorphism 8046C > T (SNP: rs1367117) is one of the major genetic-based studies that has been carried out worldwide, and it encounters strong influence over the plasma of LDL cholesterol concentrations. A population-based study on the Shanghai (Chinese) groups consisting of ~2,000 and more cumulative subjects having gallbladder, extra hepatic bile duct, ampulla of Vater cancer, and biliary stone defects was compared for their plasma lipoprotein levels with the control groups. The study also concluded that the homozygous C allele of the polymorphism is strongly associated with ~1.5-fold higher risks for bile duct related cancers and was seen predominant for 8046C > T among the two major haplotypes (64).

Similarly, two studies insisting over the multiple genetic determinants screening study associated with that of plasma lipid levels in the Caribbean Hispanic population consisting of ~477 subjects and Chinese Shanghai population comprising of 799 randomly selected healthy subjects showed that the 114 and 21 SNPs screened, respectively, related to lipid metabolism events and revealed three genes including *APOB* rs1367117 had strong influence over the HDL-C/TG, TC, and LDL-C level variations (65, 66). Later, a study on the Young adult subjects (*n* ~1,250) belonging to the Jerusalem population also showed that the *APOB* (rs1367117) polymorphism have a significant maternally-derived effect on body mass index (BMI) and waist circumference (WC) parameters providing evidence that the *APOB* gene polymorphism 8046C > T (SNP: rs1367117) plays a strong and effective role on regulatory mechanisms (67).

Along with this information, the recent *APOB* gene polymorphism studies report that they are strongly associated with CHD and CVD risks. In addition, a study carried out on the Pakistani Asian population including ~1,960 control and CHD subjects showed that the *APOB* gene 4181 E>K polymorphism (SNP: rs1042031) has an evident influence on the risks for developing CHD. Simultaneously, a study on the same population consisting of ~625 subjects screened for the rs1042031 genotype showed atherogenic blood lipid associations with the gene polymorphism when analyzed for the significant positive link with the genetic risk score (GRS). Similarly, an insertion/deletion (ins/del) polymorphism study carried out on signal peptide region of rs1042031 4154 G>A on exon 29 of the gene alters the binding efficiency of LDL to LDL receptor, causing an increase in LDL-c levels, thereby having higher risks of cardiovascular defective events (37, 68). The cumulative report on the Apolipoprotein A1/C3/A4/A5 gene cluster-based studies and the associated gene polymorphism studies having various effects on the wide range of medical complications are listed in Table 3.

**Table 3:** List of genetic studies related to the *APOA1, APOA5, APOC3, and APOA1/A4/A5/C3* gene cluster, presented with their mutation, population, sample size, and contributing medical risks.

**Gene**	**Mutation description**			**Population**	**Number of sample**	**Risk associated**	**Effect**	**References**
*APOA1*	Promotor Region	276G>A	AA AG GG	UAE	~160	CVD, Blood Pressure	↑Blood Pressure determinants for AA>AG>GG	(30)
*APOA1*	Promotor Region	276G>A	AA AG GG	Europe (Spain)	~50	Post prandial Lipoprotein Cholesterol metabolism	↓ Plasma Cholesterol, LDL Cholesterol, ApoB in G/G ↑Postprandial responsive increase in G/A	(31)
*APOA1*		75G>A		Southern Italy	~800	CVD	↑A/A ↓G/G ↑LDL Cholesterol	(52)
*APOA1* *APOB*		ApoA1/ApoB		Chinese (Han/Jing ethnic groups)	~1,869	CHD	(G-C-G-C-T-G-C-C-A) Genotype – ↑TG,TC,LDLC in Jing alone	(60)
*APOA1* *APOB*		ApoA1/ApoB		Chinese (Han/Jing ethnic groups)	~1,640	CVD	(CAPN3- FRMD5 T-A, T-C, C-A, C-C) carriers ↑TG,TC,LDLC, HDLC	(60)
*APOA1/A4/A5/C3* gene cluster		8 SNPs		English Families	~200	CVD	↑TG, LDL in FCH families for *APOA5* SNPs	(40)
*APOA1/A4/A5/C3* gene cluster		APOA556C>G C3386C>G		Dutch Families	~126	CVD	↑TG and LDL levels	(41)
*APOA1/A4/A5/C3* gene cluster		3'UTR SstI		Sri-Lankan Men	~95	CHD	↑TG	(42)
*APOA1/A4/A5/C3* gene cluster	Promotor Region	56C>G 1131T>C		American men and women	~1,200	CVD	*APOA5 -*↑TG, RLP and VLDL	(49)
*APOA1/A4/A5/C3* gene cluster	Promotor Region	1131T>C 455T>C		Korean	~158 (Non-obese)	CVD	No association	(50)
*APOA1/A4/A5/C3* gene cluster	91 SNPs in 25 Genes related to Lipoprotein metabolism			Caucasian	~688	TG, VLDL, CVD	*APOA5*_S19W show ↑TG, ↑VLDL	(32)
APOA1/B		SRGAP2		Pakistan (Kazakhs, Uighurs)	~448	↑CHD in Kazakhs	↓ApoA1 ↑ApoB Causes CHD Risk	(62)
APOA1/B		ApoA1/ApoB		Chinese	~2,444	↑CVD, CHD, Breast cancer in Han nationals	↑ApoA1/ApoB ratio	(63)
*APOA5*	Promotor Region	1131T>C	Ser>Trp Ser/Ser Ser/Trp Trp/Trp	Czech	~2,500	TG	↑Ser/Trp ↑Trp/Trp	(33)
*APOA5*	Promotor Region	1131T>C	Ser>Trp Ser/Ser Ser/Trp Trp/Trp	Europe (Spain)	~51	Choleterol, TG, ApoB100	CT/TT ↑ Postprandial TG, TRLC, TRLTG, TRLRP	(34)
*APOA5*	Promotor Region	1131T>C	Ser>Trp Ser/Ser Ser/Trp Trp/Trp	Asian women	~973	Dyslipidaemia	↑TG, TC, HDLC for the C allele	(48)
*APOA5/APOC3*		482T>C 3238C>G Signal peptide		Czech	~285	Postprandial plasma APOC3 and TG levels	RLPTG are strong predictors of atherogenity	(38)
*APOB*		8046C>T	CC CT TT	Chinese (Shanghai)	~2,000	GI complications	C allele carries >1.5% risk for bile duct cancer	(64)
*APOB*		8046C>T	CC CT TT	Jerusalem (Young Adults)	~1,250	Regulatory mechanisms	ApoB>C allele	(67)
*APOB*		4181E>K		Asia (Pakistan)	~1,960	CHD	4181E>K - ↑TG, TC, HDLC	(68)
*APOB*		4154G>A	GG GA AA	Asia (Pakistan)	~625	Binding efficiency of LDL to LDL receptors modified	TG, LDL alterations	(37)
*APOC3*		3'UTR SstI		Framingham offsprings	~5124	CHD	No significant relatedness for the S2 polymorphism	(36)
*APOC3*		T2854G	TT TG GG	Korean Men	~263	ApoC III links Postprandial TAG	↑Fat meal GG>TT>TG for TAG Risks	(37)
*APOC3*		SstI 455T>C 488C>T	rs5128 rs2854116 rs2854117			CHD	↑Fasting TG for SstI ↑Longevity and HDL for 455T>C ↑TG for 488C>T	(39)
*APOC3*		-		Triethnic groups (African-Americans) Men	~168	CVD	Apo-C(III) linked to the TG, HDL levels ↑ Metabolic syndrome	(43)
*APOC3*		455T>C T625del C3238G 482C>T		European	~500	NEFA, C/T >CHD Diabetes	455T>C, CC- CHD risk 482T/T- ↑ insulin	(51)

### Apolipoprotein E

**Figure 3:**
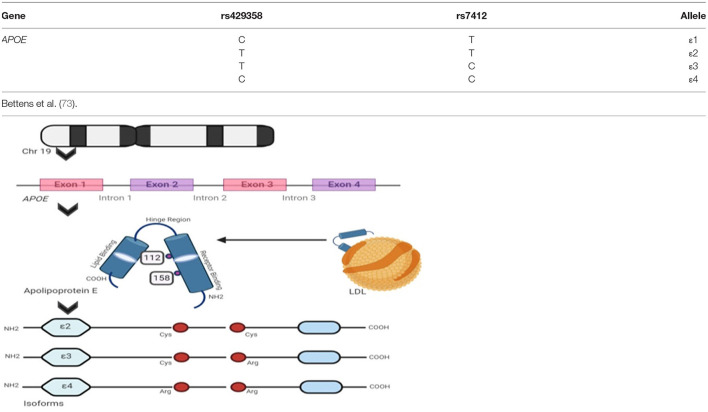
Apolipoprotein E isoforms and its genetic integrity: the figure shows *APOE* allelic variants encoded by the *APOE* gene that transcribes Apolipoprotein E isoforms and the table lists all the major genotype specific single nucleotide polymorphism (SNP) variants and their nucleotide transformations seen, respectively.

**Figure 4:**
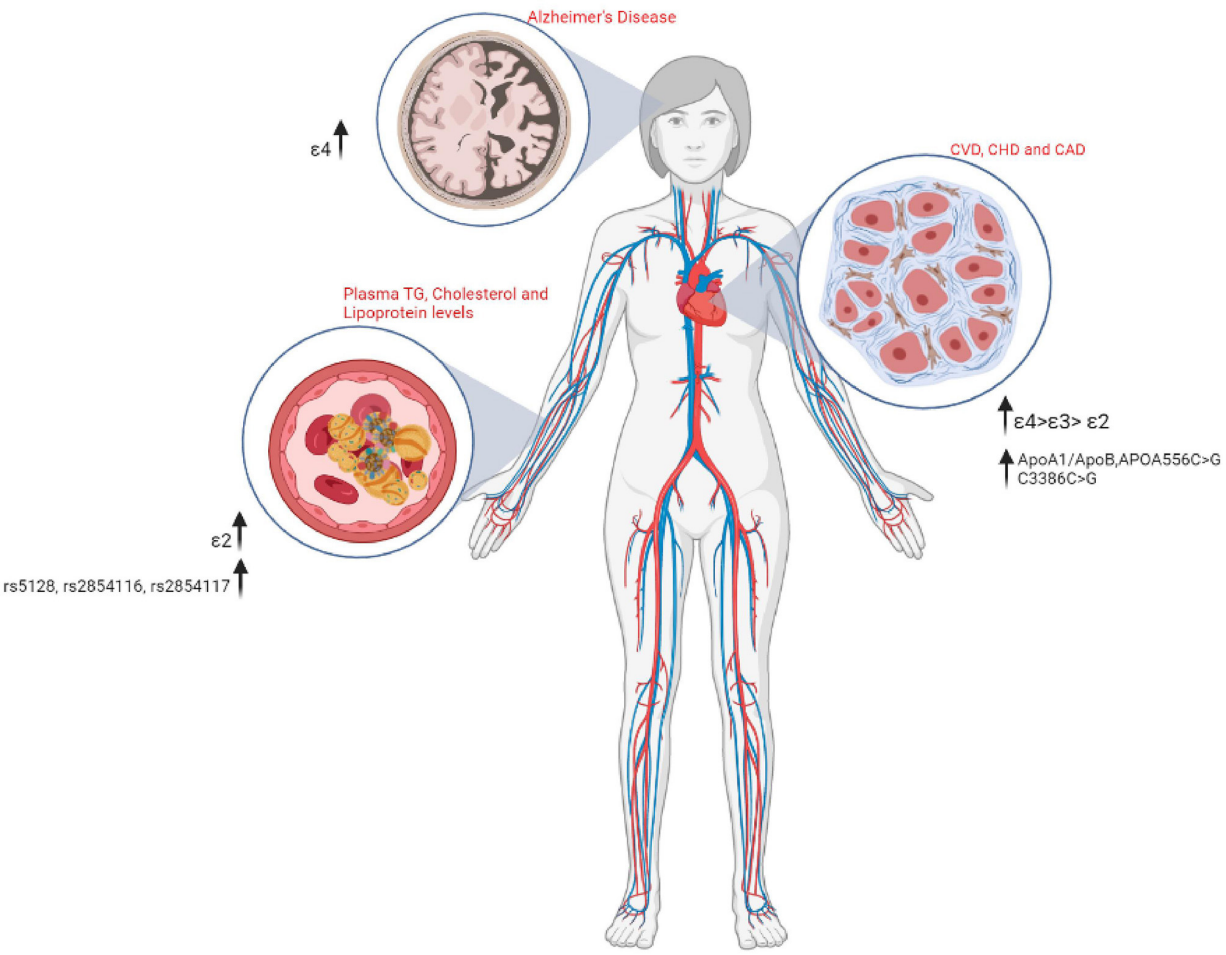
A schematic representation of the various Apolipoprotein polymorphisms and their associated medical risks.

Apolipoprotein E or APOE is a 34 kDa (kilo Dalton) protein comprising 299 amino acid residues that transport lipid molecules (69). The protein is encoded by the *APOE* gene (OMIM: 107741) located in chromosome 19, having 4 exons (70). The *APOE* gene variants generate from the exon 3 region contributing to the rs7412 C/T and rs429358 C/T, SNPs generating: ε2 (*APOE2*), ε3 (*APOE3*), and ε4 (*APOE4*) (71, 72) major haplotypes as shown in Figure 3. These isoforms ε3 have cysteine and arginine residues in 112th and 158th positions, respectively, and ε4 has two cysteine and two arginine residues, respectively (74). It contributes to six major genotypes, such as ε2/ε2, ε3/ε3, and ε4/ε4 that are homozygous forms and ε2/ε3, ε3/ε4, and ε2/ε4 heterozygous forms. Over the years, it has been strongly believed that the ε4 allelic expression in a population is the most prevalent modulator associated with AD risk. A cumulative correlation of APOE polymorphisms and associated risks are tabulated in Table 4. The clinical characteristics and biochemical associations witnessed for various APOE polymorphisms are depicted in Figure 4 respectively.

**Table 4:** The *APO*ε isoforms related genetic studies depicting the risks of various medical conditions.

**Population**	**Type of subject**	**Sample size “n”**	**Medical**	**Risk**	**Allele**	**References**
African (Senegalese)	25–80 years	>500	CAD, CVD, AD	↑	ε3/ε4	(75)
European and Chinese	Middle aged men	~100	CHD	↑	ε3, ε4	(76)
Danish and Finnish	25–80 years	~1,000	Heart attack survivors	↑	ε3, ε4	(77)
African (Malagasy)	25–80 years	~110	CAD, CVD, AD	↑	ε3, ε4	(78)
African (Tswann)	25–80 years	<100	CAD, CVD, AD	↓	ε3, ε4	(79)
African (Haoussa‘s of Niger)	25–80 years	>150	CAD, CVD, AD	↑	ε3, ε4	(80)
Australian	Post mortem analysis	~25	APOE Genotyping	↑	ε3	(81)
European	Post mortem analysis	1,500	CVD	↑	ε3, ε4	(82)
American	19–59 years	33	Plasma inflammatory markers	↑	ε4>ε3> ε2	(83)
African (Zambian)	25–60 years	116	CAD, CVD, AD	↑	ε2>ε3>ε4	(84)
Italy, Spain, Japan (European, Asian)	Old aged with younger control groups	~1,500	Longevity of age	↓	ε4	(85)
European	Old aged men	~100	Plasma TG and TC Levels	↑	ε2	(86)
Ashkenazi Jewish	~95 years	950	Dementia	↑	ε4	(87)
Brazilian	~72 years	452	Plasma TG -Stroke risk	↑	ε4	(88)
Chinese	~40–85 years	1,273	Serum Lipid levels and cognitive impairments	↑	ε3	(89)

*↑ - Increase, ↓ - Decrease*.

*CHD, coronary heart disease; CVD, cardiovascular D; CAD, coronary artery disease; AD, Alzheimer's disease; TG, triglycerides; TC, total cholesterol*.

### *APOE* and Dementia

Dementia is a generic term for memory, language, problem-solving, and other thinking abilities that are bad enough to cause daily difficulties (90, 91). Heterozygous recessive inheritance of the ε4 allele is associated with increased genetic susceptibility to the risk of dementia that ranges from an ~3 to ~15 increase in heterozygous and homozygous individuals, respectively (73, 92, 93). The ε4 allele is also associated with AD, Aortic valve disease (AVD), CAD, LDL-C levels, and longevity with the *APOE* isoforms are portrayed on a stepwise increase in risk from ε2>ε3>ε4. The strongest evidence for the role of *APOE-*ε4 isoforms in non-AD neurodegenerative diseases can be mapped to dementia with Lewy bodies (DLB) as in the case of Parkinson's disease (PD) (94).

The common *APOE* alleles: ε2, ε3, and ε4 are predominantly located in the CpG islands and these genotype expressions analyzed by means of the three alleles have an impact on the magnitude of the CpG di-nucleotides, contributing to the expression of the DNA methylation of respective gene (95). A study on DNA methylation profiles of these genomic regions assists to play a major role in differentiating the AD brain defects from control brains (96), and several other studies conducted on the African-American population suggested that DNA methylation screened in blood cells is a clear indicator of risks for developing dementia which may, later on, lead to increased risks for AD pathologies. The results showed that the methylation seen in eight CpGs demonstrated 8.7% of gene variance associated with memory defects within the APOE ε4 carriers (97). Whereas, the genotyping analysis study on post-mortem brain hippocampus and cerebellum reports of pathologically confirmed AD cases consisting of ~25 AD and control groups showed the presence of soluble protein fragments of *APOE* in *APO* ε3 expressing individuals, measuring 2- to 3-folds higher than that of the *APO* ε4 subjects (81). More significantly, a study that included 116 subjects between the age groups of 25–60 years belonging to 72 ethnic groups found in the native African population analyzed for the associations of CAD, CVD, and AD complications showed that the APOE isoform (ε4 carriers) recurrences inferred for the Zambian tribe's ancestry varied significantly between Zambian and the North African populations, such as Moroccans and Tunisians. This study suggested that the variation may be due to the extensive intermixing of the North African populations with Arab, Jewish, and other Western Eurasian populations, as reported by Wozniak et al. (78). Comparatively analysis carried out on the Zambian and other African populations (Tswana, Botswana) for the APOE allele frequencies showed 7 and 11.1% in Haoussa's of Niger and 4% (Malagasy) to 34.4% (Senegalese), respectively, as of ε2 and ε3 alleles.

### *APOE* and Vascular Complications

Some of the diseases affecting the circulatory system, such as CVD, are major causes of morbidity and mortality worldwide. An accurate prediction and screening of the genetic predisposition of an individual will incur the risks of an individual being affected by such genetic alterations. More importantly, the risk for CVD is determined by a combination of modifiable and non-modifiable genetic risks. It would be embarrassing if these reasons were not rectified and met in advance. The genetic factors involved in lipid metabolism playing key roles in CVD risks presenting increased susceptibility for the disease are accurately predicted beforehand. The genetic susceptibility for E4 allele occurrence and its strong associations with higher LDL-C concentration and total cholesterol were some of the evident initial research outcomes (98). These correlations were then studied with reference to CVD risks later, but as days scored, the association of the E4 allele with the risk of dementia was more evident in various populations around the world, making it less observed for LDL-concentrations and associated vascular complications (99). The APOE-based genetic polymorphisms are also strongly associated with plasma lipid profiles that correlate to increased risks of vascular complications in later stages of life, but the APOE polymorphisms are directly correlated with CVDs, aging, and longevity in humans (100).

A report on nine ethnic groups of the Asian population (eight European and one Chinese ancestry), such as middle-aged men, showed that variations in relative frequencies of ε4 allele occurrence could predict 40–75% risks for CHD (76). Similarly, a follow-up study on the Scandinavian population belonging to the Northern European regions that included 1,000 Danish and Finnish heart attack survivors report denoted the risk for carrying ε4 variant is one of the prognostic elements for CVD risks, as these subjects show an 80% increase risk for recurrences and death (77). Similarly, the inference presented by a pilot study that included the post-mortem analysis of 1,500 individuals who died of natural causes, performed at the Oslo University Hospital (Norway) showed that the group of patients presenting the risks of CVD (35% of the total), were significantly more susceptible for ε4 homozygous cohorts (34% against 29%) than the rest of the group having lesser chances of ε2 homozygous or heterozygous carriers (12% against 14%) for chosen *p*-values (*p* < 0.05) (82). The ε4 allele frequency between the Zambian and other African populations was as low as 1.8% in Zambian, and whereas for the Tswana population, it was as high as 23.7%. The difference between the Zambian and Senegalese populations showed 13.8, 59.5, and 26.7% for the ε2, ε3, and ε4 allelic frequencies, respectively. The ε4 allele frequency was depicted to be lower in patients with CVD, CHD, and CAD comparatively from various studies (75, 78–80, 84).

The transport and clearance mechanism of chylomicron remnants, VLDL, and HDL from the bloodstream is brought about by the action of the *APOC3* gene (57). The −482 C/T (rs2854117) and −455 T/C (rs2854116) polymorphisms of APOC3 are actively associated with non-alcoholic fatty liver disease (NAFLD) and insulin resistance (IR) (101). Sometimes, it is also considered that the ApoCIII promotes hypertriglyceridemia (HTG), inhibits the activity of LPL, disturbing lipids lipolysis, and interferes with apoE or apoB binding to hepatic receptors causing delayed catabolism of TRL remnants, regulates the VLDL assembly, and secretion in the liver through different mechanisms (102, 103). In such cases, the ApoCIII and lipoproteins that carry apoCIII are studied as the key regulators in the TG metabolism and independent predictors of CVD risk. Since the APOC3 brings in a phenotypic change in the effect on lipid metabolism, it is also believed to contribute to an increased risk of CAD (104). Similarly, the APOA5 plays a key role in modulating TG metabolism, thereby acting as a potential regulator of plasma TG causing CVD risks (105).

### *APOE* and Longevity

A similar pattern of study provoked from the highlighted presumed associations of *APOE* and extreme lifespan longevity focused on three independent cohorts of older aged groups from Italy, Spain, and Japan, compared for healthy and younger controls. This study confirmed that the occurrence of the ε4 allele has a negative association with that of the extreme longevity in all three cases after adjustment for gender based correlations. This served to be one of the major genes based positive association studies that concluded the presence of the same traits of the ε2 allele in Japanese and Italian cohorts. This strongly highlighted that the ε4 variants validate to decrease the probability of the increasing risks of ages across ethnicity and geographic origins (85). Later, in a study on Ashkenazi Jewish ancestry subjects, ~1,000 individuals consisting of older groups having occurrences of familial longevity history (~95 years of age) and younger aged control groups were analyzed for the full-length expressions of *APOE* gene regions. The analysis showed the occurrence of two common regulatory variants (rs405509 and rs769449), playing regulatory functions *via* promoter regions of the gene. The results showed significantly depleted expressions of ε2 allele (*p* = 0.003) and the ε2/ε3 genotype (*p* = 0.005) in the elderly groups for the designated *p* values (*p* <0.036) and evident amelioration of the ε3/ε4 genotype (*p* = 0.005) when compared with the control groups seen with increased longevity characteristics (87).

The APOε genotypes were not only believed to have recurrences with AD and heart diseases but have also been associated with the fluctuating levels of unsaturated and saturated circulating fatty acids (106). People who are obese possess increased blood or plasma cholesterol, glucose, and insulin conditions that lead to several other diseases, such as diabetes and other metabolic syndromes, independently associated with unique cognitive deficits (107–109). The first study reported on the Brazilian population that included 220 subjects above the age of 85 years, 232 controls averaging to the age of 72 years comprising of the oldest-olds, investigated the association among *FOXO3, SOD2, SIRT1*, and *APOE* known gene variants. The investigation showed that the only association seen for the gender-based TG levels was confirmed with that of the *APOE* isoforms ε2 and ε3 but for the rest was normal. Moreover, the report also suggested that expansion of samples size with gender- and age-based criteria for the chosen population would yield better confirmative results (88).

### *APOE* and Obesity

In the European population, a study conducted for analyzing the small portion of ε2 allele variant, the rarest amongst the six genotypes, showed that the known variant recurrences have risks for developing lipid metabolisms related disorders, mainly contributing to clinical pathogenesis known as the Type III hyperlipoproteinemia that is characterized by extreme elevations of TGs and TC levels.

Several studies suggest that the occurrence of the ε2 homozygous genotype has higher risks for CAD, palmar xanthomata (orange creases in the palms), and tuberous xanthomas (nodular lipid deposits) on skin, knees, and elbows (86). In addition, the ε2 allele does not appear to be protective against ischemic stroke (IS) as reported by the study that was carried on European subjects aged above 75 years with recurrent cardiovascular events (110). Whereas in terms of the APO ε4 allele expressions, a study reported with the Chinese population consisting of 1,273 subjects (aged between 40 and 85 years) analyzed for APOE genotype and concordant Serum lipid levels showed no significant correlations between the serum LDL—TG/TC levels and the APO ε4 allele expressions having the odds ratio (*OR*) of *OR* = 20.094 for *OR*@95% with the estimated *p*-value of 0.039 (*p* ≤ 0.005). The study reported that the homozygous ε4 carriers have lower serum HDL with positively concordant cognitive impairments, showing that the APOE isoforms play major roles in serum lipid levels and the assessments of cognitive impairments and memory (89).

Moreover, the APOE isoforms are believed to play a key role in modulating the immune responses of inflammatory mediators secreted by the microglia cells through HDL and LDL levels of the brain in an APOε4>APOε3>APOε2 pattern (111). Where obese patients present increased activated macrophages and pro-inflammatory cytokines, tumor necrosis factor-α (TNF)-α, and interleukin (IL)-6 secretion from adipose tissue. Moreover, APOE isoforms are directly linked with the expression of the plasma inflammatory markers where the exogenous expression of APOε3 acts as a critical suppressor of inflammatory processes, and the APOε4 expressions are linked with overexpression of the pro-inflammatory TNF-α, IL-1β, IL-6, and IL-12 immune cells being major phenotype markers of JNK, MAPK p38, and casein kinase 2 (CK2) pathways (112). APOE genotype study carried out on 33 middle aged cohorts (19–59 years of age) of the American population related to the plasma signaling protein levels showed a concomitant expression of the inflammatory markers I-309, IL-1, IL-3, IL-7, IL-12p40, IL-13, and IL-15, to be the lowest in ε2 allele followed by the APOε3>APOε4 patterns (83).

The effects of APOE genotype variants in obese populations are based on several phenotypical features, such as sex, age, and co-inherited gene variants. The E4 midlife obese carriers showed increased susceptibility for AD risks in later stages of life, whereas the increased waist to hip ratios were associated with impaired cognitive deficits in middle-aged cohorts expressing increased white matter hyper-intensities (113, 114). Similarly, weight loss in older Prospective Population Study of Women (PPSW) in Sweden showed an increased risk of cognitive decline concerning declined BMI and E4 allele recurrences (115). The mechanism of cellular obesity occurring during the middle-ages is strongly associated with decreased blood-brain barrier (BBB) integrity, tau phosphorylation, and decreased amyloid precursor protein (APP) levels. The gender-based susceptibility of AD risk is higher in the case of obese women presenting deficits in BBB integrity, and high BMI associated inflammation at an older age (116, 117). More interestingly, the APOE4 carriers are comparatively more sensitive to metabolic alterations associated with obesity (118). Apart from which the recycling mechanism of APOE appears to depend on the surface expression of LDL receptors and ATP binding cassette 1 (ABCA1) activities. The E4 carriers are strongly associated with lower BMI but increased susceptibility to higher insulin associated metabolic syndromes (119).

### *APOE* and Immunity

The accumulation of pathological proteins in AD brains, being a prominent feature, induces the activation of glial cells, leading to prolonged neuro-inflammation that might induce neuronal injury or death in multiple ways (120). This cascade reaction involves the initial activation of glia producing toxic compounds that fall under two major categories, i.e., the reactive oxygen species (ROS) or nitric oxide (NO) that damage neurons. Sometimes, this reaction is also replaced by the sublethal pathological inflammatory stress that can damage viable neurons to express apoptotic signals that cause phagocytosis of the damaged neurons through a process called phagoptosis (121). The neuro-inflammation process is the major hallmark that contributes to the onset and progression of AD pathogenesis. The activated microglia responded to the pro-inflammatory mechanisms through the release of cytokines, such as TNFα and interleukin 1β (IL-1β), additionally inducing damage to other surrounding glial and anchoring cells (122).

The expression of APOE suppresses the proliferation of T cells and activation of a neutrophil by regulating and facilitating macrophage functions and lipid antigen presentation by the CD1 molecules. Naturally, the cellular ApoE regulates the lipopolysaccharides (LPS) induced lethality by attenuating inflammatory responses, where deficiency of ApoE causes impaired immune responses leading to the production of significantly higher pro-inflammatory cytokines, such as IL-1, IL-6, and TNF- (123). On the other hand, the ApoE suppresses the production of pro-inflammatory cytokines, through a descending order as E2>E3>E4 isoforms. The crucial initiation process of the innate and adaptive immunities relies on macrophage activation, and this is achieved by the classical activation of the inflammatory stimuli *via* the pro-inflammatory signals that are accompanied by the downregulation of ApoE expression. One of the conventional identities of activated macrophages is observed by the production of NO. Several animal studies on Human TG mice show that the increased ApoE treatment induces the production of macrophages and microglia with higher NO secretion. Recent studies also show that the B cells utilize ApoE-mediated pathways for lipid antigen presentation rather than the dendritic cells and these ApoE based CD1-mediated self-lipid presentation to NK-cells are widely involved in autoimmune diseases, such as atherosclerosis, multiple sclerosis, and others (124, 125).

### *APOE* and Stroke

A stroke is a form of disability, characterized by the blockage of blood vessels causing detrimental effects that lead to the second largest form of death occurring in the older population worldwide (90, 126–129). The age-based risk for stroke increases with age and is doubled over the age of 55 years in both men and women (130, 131). Some of the subtypes of stroke, such as the lobar intracerebral hemorrhage, are associated with an increased risk on the occurrences of ε2 or ε4 homozygous alleles, where the *ApoE*ε4 allele carriers are reported with higher volumes of subcortical white matter lesions presenting increased risks for IS and associated medical complications (132).

The *ApoE* ε4 allele carriers were said to exhibit significant risk for IS, and an increased risk of ICH was associated with the presence of ε2 allele by a meta-analysis report in 2006 (133). The meta-analysis reports from Zhang et al. (134) showed that the ε4 allele carriers present increased risks for ICH but not the ε2 allele carriers. But the actual association of the ApoE polymorphism and IS or ICH in the Chinese ethnic population is still an outstanding question. Similarly, a meta-analysis study carried out later on 18 different reports that have worked in various Chinese provinces suggested that there is a significant association between the ε4 allele carriers and increased ICH risk when compared with that of the ε3 and ε2 alleles. Similarly, the study showed a significant association for ε4 carriers and ε3ε3 genotype carriers in IS risk with an *OR* of 2.41 and 2.00–2.89 for (95% *CI* at *p* < 0.001) (135). A study reported on South Indian population also showed that the frequency of HLA-DRB1/DQB1alleles were higher in IS patients but not in control, predicting DRB1*10-DQB1*05 haplotype existence to be protective over IS (136).

## Conclusions and Future Perspectives

The current review has put forth the progressive population genetic data that have been great insights in understanding the role of genetic polymorphisms that are correlated to several APO protein-based medical risks. From the cumulative knowledge perceived, the contribution of genetic polymorphisms that influence the lipoprotein levels appear to be associated with selective risks, such as cardiovascular associated disorders, neurodegenerative disorders, metabolic syndromes, and other lipidaemias, respectively, across several population groups. From future perspectives, the comprehensive understanding of such molecular events correlated with several environmental and dietary factors can unravel novel perceptions of various therapeutic approaches interlinked to the abovementioned clinical manifestations. In addition, the review strongly suggests that a broad range of molecular analysis studies considering factors, such as patient's history of ethnicity and habitual traits with a huge sample size across various populations will be a challenging field to entangle in the near future, and this can further develop the understandings of unspotted haplotype-based studies and narrowing down the complications in several metabolic disorders associated risk assessments.

## Author Contributions

RB: raw data and basic information collection. PB: conceptualization and writing—original draft preparation. DK: data curation and formal analysis. VP: guidance, validation, and supervision. All authors contributed to the article and approved the submitted version.

## Conflict of Interest

The authors declare that the research was conducted in the absence of any commercial or financial relationships that could be construed as a potential conflict of interest.

## Publisher's Note

All claims expressed in this article are solely those of the authors and do not necessarily represent those of their affiliated organizations, or those of the publisher, the editors and the reviewers. Any product that may be evaluated in this article, or claim that may be made by its manufacturer, is not guaranteed or endorsed by the publisher.

## Abbreviations

APO proteins: Apolipoproteins
SNP: single nucleotide polymorphism
CVD: cardiovascular disease
AD: Alzheimer's disease
HDL: high-density lipoprotein
IDL: intermediate-density lipoprotein
LDL: low-density lipoprotein
VLDL: very low-density lipoprotein
CHD: coronary heart disease
PVD: peripheral vascular disease
APOE: Apolipoprotein E
APOA: Apolipoprotein A
APOB: Apolipoprotein B
APOC: Apolipoprotein C
CAD: coronary artery disease
PD: Parkinson's disease
DLB: dementia with Lewy bodies
LDL-C: low-density lipoprotein-cholesterol
AVD: aortic valve disease
CK2: casein kinase 2
Lp (a): lipoprotein (a)
TG: triglycerides
FCH: familial combined hyperlipidemia
HDLC: high-density lipoprotein cholesterol
GHD: growth hormone deficiency
TC: total cholesterol
IRE: insulin responsive element
UTR: untranslated region
NEFA: non-esterified fatty acid
LCAT: lecithin cholesterol acyltransferase
PCSK9: proprotein convertase subtilisin/kexin type 9
DOCK7: dedicator of cytokinesis 7
GRS: genetic risk score
UAE: United Arab Emirates
RLP: remnants of lipoprotein elements
ROS: reactive oxygen species or nitric oxide (NO).
